# Proteolytic cleavage of Slit by the Tolkin protease converts an axon repulsion cue to an axon growth cue *in vivo*

**DOI:** 10.1242/dev.196055

**Published:** 2020-10-29

**Authors:** Riley Kellermeyer, Leah M. Heydman, Taylor Gillis, Grant S. Mastick, Minmin Song, Thomas Kidd

**Affiliations:** Department of Biology/MS 314, University of Nevada, 1664 North Virginia Street, Reno, NV 89557, USA

**Keywords:** Axon guidance, Proteolysis, Robo, Slit, Slit fragments, Tolkin tolloid-related

## Abstract

Slit is a secreted protein that has a canonical function of repelling growing axons from the CNS midline. The full-length Slit (Slit-FL) is cleaved into Slit-N and Slit-C fragments, which have potentially distinct functions via different receptors. Here, we report that the BMP-1/Tolloid family metalloprotease Tolkin (Tok) is responsible for Slit proteolysis *in vivo* and *in vitro.* In *Drosophila* *tok* mutants lacking Slit cleavage, midline repulsion of axons occurs normally, confirming that Slit-FL is sufficient to repel axons. However, longitudinal axon guidance is highly disrupted in *tok* mutants and can be rescued by midline expression of Slit-N, suggesting that Slit is the primary substrate for Tok in the embryonic CNS. Transgenic restoration of Slit-N or Slit-C does not repel axons in Slit-null flies. Slit-FL and Slit-N are both biologically active cues with distinct axon guidance functions *in vivo*. Slit signaling is used in diverse biological processes; therefore, differentiating between Slit-FL and Slit fragments will be essential for evaluating Slit function in broader contexts.

## Abstract (precis)

**Summary:** The axon repellent Slit is proteolytically cleaved by Tok and the resulting Slit-N fragment promotes longitudinal axon growth, not repulsion, *in vivo*.

## INTRODUCTION

Navigating axons respond to extracellular cues for directionality and survival. These extracellular cues instruct axon attraction, repulsion, growth, adhesion and apoptosis. The complexity of the nervous system is generated by a remarkably small number of cues. Some diversity in the function of cues can arise from distinct receptor complexes, tight regulation of receptor localization in the growth cone via intracellular trafficking (Stoeckli, 2018), or by modulating the strength and duration of signaling through receptor proteolysis (Bai and Pfaff, 2011). Ligand proteolysis offers an additional area of potential signaling regulation or diversification. For example, neurotrophic factors are synthesized as pro-neurotrophins that can trigger cell death but switch to promote neuronal survival after proteolytic cleavage (Costa et al., 2018). In this article, we demonstrate how proteolysis of the axon guidance ligand Slit generates fragments with *in vivo* activities that oppose those of the full-length Slit protein (Slit-FL), ultimately diversifying the signaling output of a single guidance cue.

In the central nervous system (CNS) midline, axons are directed to cross or grow longitudinally adjacent to the midline by a small set of signaling molecules (Comer et al., 2019; Howard et al., 2017). The large secreted protein Slit plays an important role in repelling axons from the midline, acting through Roundabout (Robo) receptors (Bisiak and McCarthy, 2019; Brose et al., 1999; Kidd et al., 1999, 1998). Slit-Robo signaling can be controlled through receptor function, notably with Robo receptors requiring proteolytic cleavage by the protease Kuzbanian to fully function (Coleman et al., 2010). In addition to receptor-mediated regulation, the Slit signal can be altered by proteolytic cleavage that generates two functional N-terminal and C-terminal fragments (Slit-N ∼140 kDa and Slit-C ∼55 kDa; Brose et al., 1999). This proteolytic process provides a system that can regulate both signal termination and signal diversification. Slit-N was previously suspected to be the only biologically active fragment of Slit, because it includes the Robo-binding site and most Slit functions are Robo dependent (Ordan and Volk, 2015). However, significant evidence in multiple systems suggests that Slit-FL, Slit-N and Slit-C are all necessary for normal development (Battye et al., 2001; Chen et al., 2001; Morlot et al., 2007; Coleman et al., 2010; Delloye-Bourgeois et al., 2015; Svensson et al., 2016; Caipo et al., 2020).

Although both Slit-FL and Slit-N bind Robo receptors, Slit-FL and Slit-N are functionally distinct and possibly antagonistic in several biological settings, and Slit-C has Robo-independent functions. Loss of all *slit* activity in *Drosophila* leads to a dramatic phenotype in which all CNS axons collapse onto the midline (Rothberg et al., 1988). The midline collapse of axons in *slit* mutants is specifically attributed to the loss of Slit-FL/Robo signaling. Genetic rescue experiments using an uncleavable *slit* transgene (Slit-UC) demonstrated that full-length Slit is sufficient for midline repulsion (Coleman et al., 2010). Longitudinal axon guidance was not rescued by Slit-UC, suggesting a specific role for Slit-N or Slit-C in longitudinal axons (Coleman et al., 2010). However, longitudinal axons were also not rescued by expression of cleavable Slit-FL, likely because accurately replicating endogenous levels of Slit in rescue experiments is challenging (Battye et al., 2001; Kidd et al., 1999). Further studies indicated that Slit-N aids in the proper formation of longitudinal axons, as Slit-N, but not Slit-FL, binds the Down Syndrome cell-adhesion (Dscam) receptor (Dascenco et al., 2015), and forms a complex with Robo1 and Dscam1 that is required for longitudinal axon growth (Alavi et al., 2016). This latter work provides a molecular explanation for how Slit-FL and Slit-N could have different signaling outputs, via alternative receptor complexes, and establishes longitudinal axon guidance as a biological readout for Slit-N functions, independent of Slit-FL-mediated midline repulsion. Additionally, Slit-N and Slit-FL are incapable of substituting for one another in fly muscle development and optic lobe formation (Ordan et al., 2015; Caipo et al., 2020). No functions for Slit-C have been yet identified in *Drosophila*.

Differences in Slit fragment function extend to vertebrate systems. Slit2-N was biochemically purified as a factor that promotes axon branching of dorsal root ganglia, whereas Slit2-FL or uncleavable Slit2 antagonizes this activity (Nguyen Ba-Charvet et al., 2001; Wang et al., 1999). Slit2-FL and Slit2-N can elicit either overlapping or contrasting responses in other axon systems, possibly dependent on axon type and receptor availability (Ma and Tessier-Lavigne, 2007; Nguyen Ba-Charvet et al., 2001). Slit2-N also promotes neuronal survival, whereas Slit-FL may activate caspases in axons (Campbell and Okamoto, 2013; Piper et al., 2002). More broadly, Slit2-N functionally opposes Slit2-FL in models of pancreatic cancer and capillary leakage during infection (Gohrig et al., 2014; Jones et al., 2008; London et al., 2010). Finally, recent *in vivo* mammalian evidence suggests that the highly diffusible Slit2-C fragment has Robo-independent functions in axon repulsion, via Plexin receptors, and can regulate thermogenesis in beige fat cells (Delloye-Bourgeois et al., 2015; Svensson et al., 2016). Importantly, the biological roles of the Slit fragments have not been separately or independently tested *in vivo*, in part due to the unknown identity of the Slit protease, which prevented separation of Slit fragment activities from that of the full-length protein.

In this article, we identify the Slit protease as the zinc metalloprotease Tolkin (Tok; also known as Tolloid-related, Tlr, or Piranha). Tok is a member of the BMP1/Tolloid family of Astacin-like metalloproteases, with established non-cell autonomous functions in longitudinal axon guidance and motor axon fasciculation (Meyer and Aberle, 2006; Serpe and O'Connor, 2006). We find that Tok is necessary for Slit proteolysis *in vivo*, and is necessary and sufficient for Slit proteolysis *in vitro*. Tok is expressed at the right time and place during embryonic development to cleave Slit for axon guidance functions. *tok* mutants have disrupted longitudinal axon guidance, but midline repulsion is unaffected because Slit-FL is still functional. This has allowed us to separate the activities of Slit-FL and Slit fragments. Slit-N, but not Slit-C, is capable of rescuing the *tok* longitudinal axon guidance phenotype, indicating that Slit is the primary substrate for Tok at the CNS midline. Additionally, neither Slit-N nor Slit-C can rescue midline repulsion in *slit* mutants. As receptor binding promotes Slit proteolysis (Ordan and Volk, 2015), and Slit-FL has a necessary midline function, we hypothesize that Slit-FL elicits a repulsive signal in the growth cone, via Robo binding. Tok-mediated Slit proteolysis can then convert repulsion into axon growth away from or adjacent to the Slit-FL source, via Slit-N/Robo1/Dscam1 complexes (see Fig. 6).

## RESULTS

### Identification of candidate Slit proteases

**Fig. 1.**
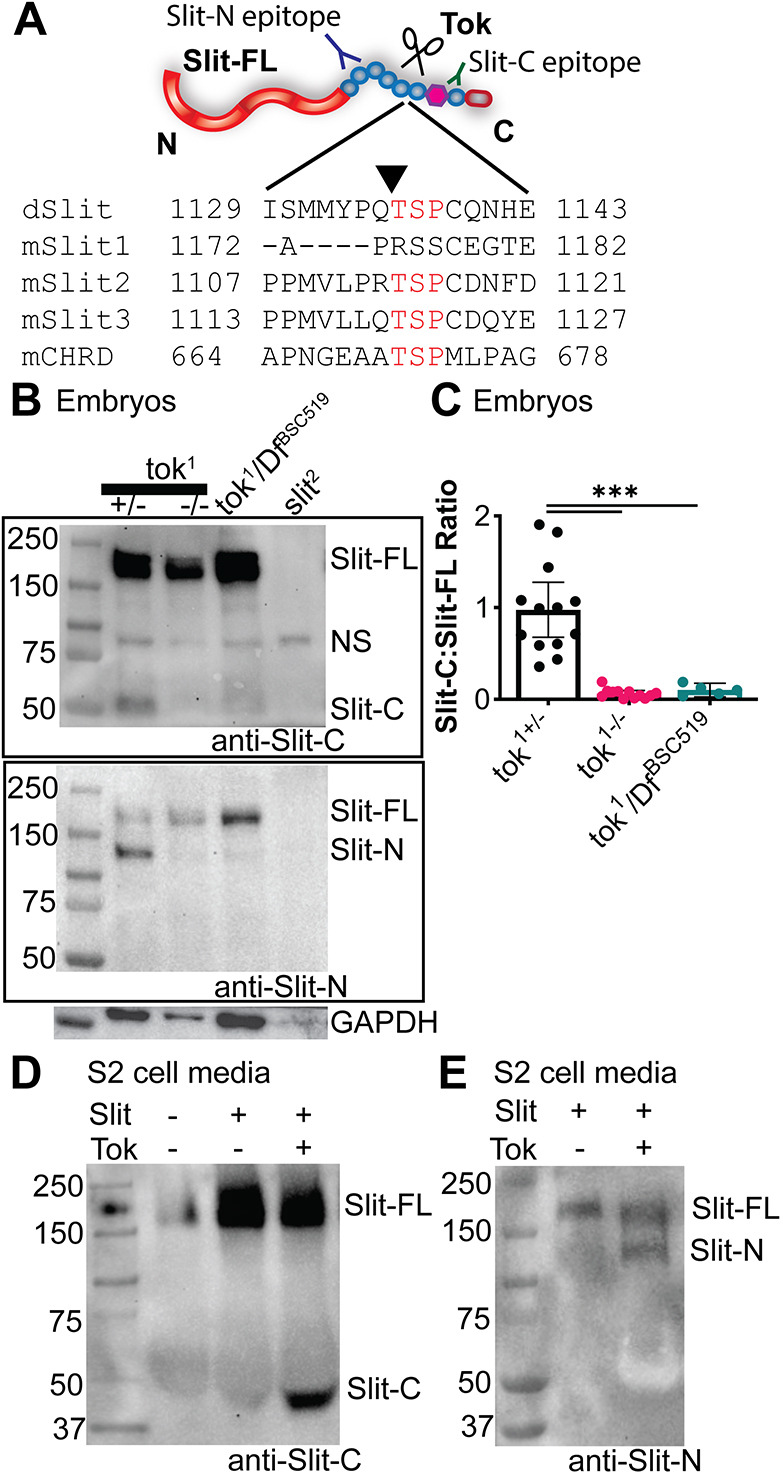
**The Tok protease cleaves Slit *in vivo* and *in vitro*.** (A) Alignment of Slit cleavage sites for fly and mouse Slit proteins showed a conserved TSP amino acid sequence between EGF5 and EGF6. This sequence is present in mouse Chordin, which is cleaved by the Tok ortholog Tolloid-like1 (Tll1). The epitopes of the Slit antibodies used in both western blot analysis and immunohistochemistry are labeled on the diagram of Slit, with anti-Slit-N specific to EGF1-3 (purple; amino acids 921-1033) and anti-Slit-C specific to the laminin G domain and EGF7 (green; amino acids 1311-1480). (B) Western blot of late-stage whole-embryo protein extractions of *tok* alleles labeled with a monoclonal antibody for Slit-C (C555.6D, top blot), which was stripped and re-probed (see Materials and Methods) with a monoclonal antibody specific to Slit-N (10B2; bottom blot). *tok^1^* homozygous mutants and transheterozygous *tok^1^* and deficiency [*Df(3R)^BSC519^*] mutants failed to process Slit, with only Slit-FL (∼180 kDa) observed and no Slit-C (55 kDa) or Slit-N (140 kDa). A non-specific band (NS) is labeled by the C555.6D Slit-C antibody (∼75-80 kDa) that is a common artifact seen in approximately half of more than 25 blots, including in *slit* knockouts. Although the *slit* knockout (*slit^2^*) had less labeling of the GAPDH loading control, the non-specific band labeled by C555.6D appeared consistent with other samples, indicating sufficient protein levels for comparison. (C) Quantification of Slit processing, measured as a ratio of relative pixels of Slit-C compared with Slit-FL, showed a significant reduction in Slit cleavage in *tok^1^* homozygotes and *tok^1^/Df(3R)^BSC519^* transheterozygotes, compared with *tok^1^* heterozygotes (***P<0.001, Welch one-way ANOVA, *n*=13 independent experiments for *tok^1^* alleles and *n*=3 independent experiments for *tok^1^/Df^BSC519^*). Data are mean±95% CI. (D) Co-transfection of S2 cells with plasmids encoding *Drosophila* Slit-FL alone showed no Slit cleavage in the media, visualized using the Slit-C antibody. S2 cells cleaved Slit when co-transfected with Slit-FL and Tok to generate a Slit-C fragment (*n*=5 independent experiments). (E) Co-transfection of S2 cells with plasmids encoding Slit-FL and Tok resulted in Slit cleavage in media, as visualized by Slit-N antibody labeling.

To identify Slit protease candidates, we looked for protease mutants that have longitudinal axon guidance defects similar to *slit*-*UC* and *Dscam1* mutants, which would indicate the absence of Slit-N/Dscam1/Robo1 complexes (Alavi et al., 2016; Coleman et al., 2010). We expected that the protease would be expressed near the midline during embryonic development, specifically during stages with the highest rates of Slit processing and Slit-N-dependent axon guidance (Alavi et al., 2016; Furrer et al., 2007). In addition, the protease would lack expression in the insect S2 cell line, which does not endogenously cleave Slit (Brose et al., 1999). Literature searches identified *tok* and other genes such as *matrix metalloproteinase 2*, as proteases with expression patterns and phenotypes that matched our predictions (Miller et al., 2008; Serpe and O'Connor, 2006). The expression of all candidate genes in S2 cells was assessed by querying the *Drosophila* RNAi Screening Center database (Hu et al., 2017), and *tok* expression was found to be absent. The cleavage sites for Slits have a conserved N-terminal TSP motif (threonine, serine, proline) in a linker region between the fifth and sixth EGF domains, although it is likely that additional flanking residues or domains are necessary. Cleavage is context dependent, because Slit1 is cleaved similarly to Slit2, but lacks the -TSP domain (Fig. 1A; Brose et al., 1999). The Cutdb database (Igarashi et al., 2007) listed the -TSP site as being a cleavage site for BMP1/Tolloid proteases in mammalian Chordin (Scott et al., 2001). These observations implicated the fly homolog Tok, which is expressed at the expected developmental time and location, as the leading candidate to be the Slit protease.

### Tok cleaves Slit *in vivo*

To test whether Tok is necessary for *in vivo* Slit cleavage, we analyzed late-stage embryos homozygous for a null allele of *tok* (*tok^1^*) and found that Slit is not cleaved in these mutants (Fig. 1B,C). A transheterozygote of this null *tok* allele with a chromosomal deletion for the region [*Df*(*3R*)*^BSC519^*] showed the same phenotype (Fig. 1B,C). A strong hypomorph *tok* allele (*tok^X2-41^*, also known as *tlr^X2-41^*) showed reduced Slit processing, but mutants for the closely related gene *tolloid* (*tld*) processed Slit normally in late-stage embryos (Fig. S1A,B). This supported the hypothesis that *tld* and *tok* evolved separately after a gene duplication event (Finelli et al., 1995; Nguyen et al., 1994). Overexpression of Tok did not appear to enhance Slit cleavage or change the overall levels of Slit protein (data not shown). This is not surprising given that Slit cleavage is tightly regulated, and previous reports showed that 92% of Slit remains in full-length form during key axon guidance stages 14-17 (Furrer et al., 2007). We were unable to replicate the lack of Slit cleavage in *amontillado* (*amon*) mutants, a proprotein convertase that was previously identified as a potential Slit protease (Ordan and Volk, 2016). We found that two independent mutant alleles of *amon* cleaved Slit normally (Fig. S1A,B). We conducted a complementation cross for lethality of these two mutant alleles (*amon^Q178st^* and *amon^C241Y^*) and found they failed to complement, confirming the presence of *amon* specific mutations. These results establish that Tok is the sole Slit protease in late-stage *Drosophila* embryos.

### Tok cleaves Slit *in vitro*

To confirm that Tok is necessary and sufficient to cleave Slit, we expressed both genes in *Drosophila* S2 cells, which make a small amount of endogenous Slit but lack Slit protease activity (Brose et al., 1999). In the absence of exogenous Tok, only full-length Slit was detected in the media of these cells (Fig. 1D,E). Co-transfection of Tok and Slit-FL induced Slit processing. Co-transfection of Tok and Slit-FL into mammalian COS-7 cells, which have endogenous Slit protease activity, also led to a significant increase in Slit processing (Fig. S1C,D). Our *in vivo* and *in vitro* results establish Tok as necessary and sufficient to cleave Slit.

### Tok and Slit interact in the extracellular space

**Fig. 2.**
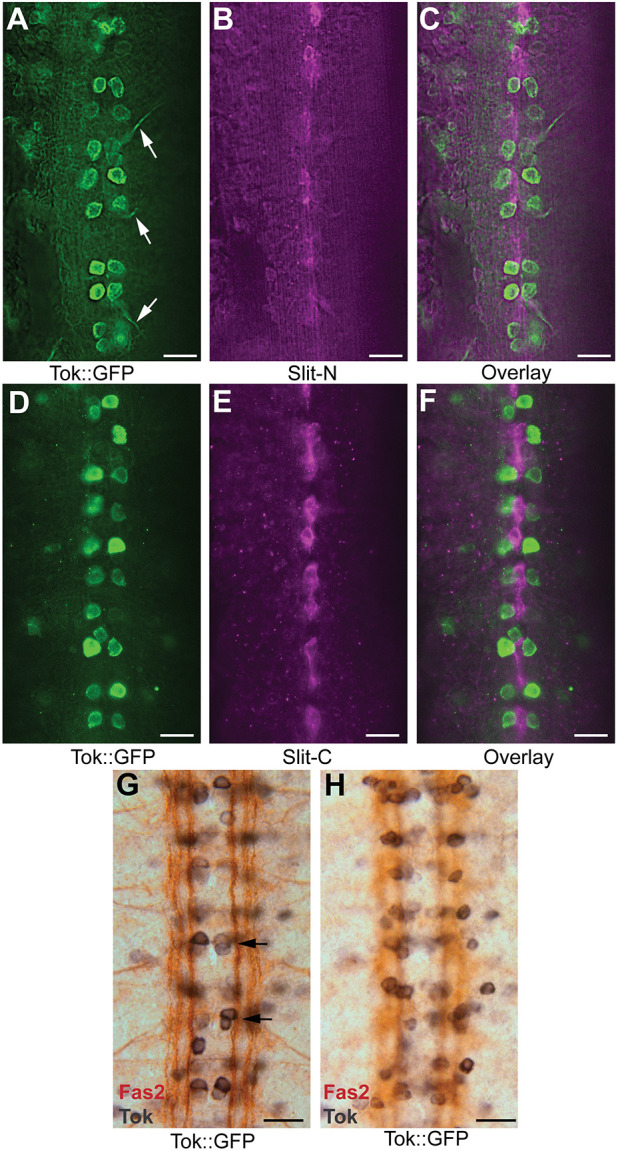
**Co-labeling of Tok and Slit showed non-overlapping expression in the CNS.** Tok expression visualized using a Tok allele with a MIMIC Trojan-Gal4 insertion driving a 10X UAS-mCD8::GFP (Tok::GFP) labeled with anti-GFP (A-F, green; G,H, black) and co-labeled with antibodies for Slit-N (A-C; magenta), Slit-C (D-F; magenta), or anti-Fasciclin 2 (Fas2; G,H; brown). Embryos are mid to late-stage 17 (A-F; *n*=3) or stage 15 (G-H; *n*=2), anterior is upwards in all images. Scale bars: 10 µm. (A) Tok::GFP expression was specific to a distinct subset of neuronal cell bodies, with Tok::GFP visible in motor axons as they exited the CNS (arrows; Movie 1). (B) Slit-N antibody labeled underlying glial cells at the midline (Movie 1). (C) Overlay of Tok::GFP and Slit-N labeling at the axon level showed little overlap. (D) Tok::GFP expression was specific to neuron cell bodies at the level of CNS axons. (E) Slit-C antibody labeled segments of midline glia at the level of the neuropil. (F) Slit-C and Tok::GFP overlay had no specific overlap. (G,H) Co-labeling of Tok::GFP using anti-GFP with nickel chloride (black) and anti-Fas2 (brown). Fas2 allows visualization of longitudinally projecting axons. (G) A segmentally repeated pair of neurons on either side of the midline resembled the aCC motor neuron and the pCC interneuron (arrows). (H) Different focal plane view of (G) showing the soma of distinct neurons expressing Tok.

After confirming that Tok cleaves Slit, we assessed their interaction *in vivo*. In *Drosophila* embryos, *tok* is expressed during gastrulation in the mesoderm (stage 6), then the midline primordium (stage 11), and subsequently in mesoderm that lies on top of the nascent ventral nerve cord (stage 12). Midline neuron expression is visible by stage 13 (Nguyen et al., 1994; Kearney et al., 2004) and Tok has strong ventral nerve cord expression from stage 14 through larval stages in a distinct subset of midline neurons (Finelli et al., 1995; Nguyen et al., 1994; Serpe and O'Connor, 2006; Wheeler et al., 2009). Slit expression starts during gastrulation and is specifically expressed by midline glia from nerve cord condensation through CNS development (Rothberg et al., 1990). Slit cleavage peaks during gastrulation (stages 6-7) and to a lesser extent during late-stage axon guidance decisions and nerve cord condensation (stages 16-17; Furrer et al., 2007). As uncleaved Slit-FL is required in midline repulsion (Coleman et al., 2010) and previous genetic rescue experiments indicate that Tok acts non-cell autonomously (Meyer and Aberle, 2006; Serpe and O'Connor, 2006), we expected Tok and Slit to have non-overlapping expression patterns in the CNS to avoid Slit cleavage before secretion. *Tok*-expressing cells were identified using a MiMIC transposon expressing GAL4 in the pattern of the endogenous *tok* locus, allowing expression of *UAS-mCD8-GFP* (*Tok::GFP*; Lee et al., 2018). Co-labeling of Tok and Slit confirmed that they were expressed by different cells, with Tok expressed by neuron cell bodies and Slit secreted by glia at the neuropil level (Fig. 2, Movie 1). To better understand which neurons express Tok, we co-labeled Tok with anti-Fasciclin2 (Fas2), which is specific to longitudinal axons. Tok appeared to be expressed by motor neurons, likely aCC or RP2 neurons, with Tok seen extending along motor neuron axons, as well as underlying cell bodies during stages 15-17 (Fig. 2A,G,H; Finelli et al., 1995; Kearney et al., 2004). Our results indicate that, in the CNS, Slit and Tok must interact in the extracellular space.

### Genetic rescues demonstrate that Slit-N functionally opposes Slit-FL

**Fig. 3.**
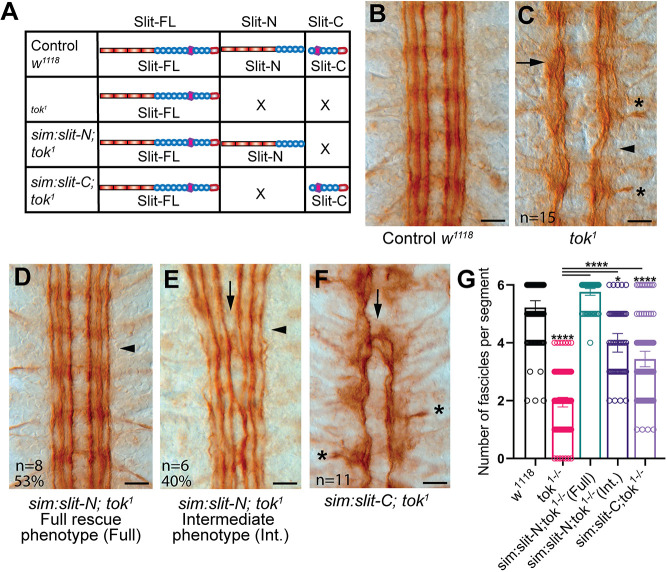
**Tok protease function is required for longitudinal axon guidance and is rescued by midline expression of Slit-N.** (A) Graphical representation of Slit fragments present in *tok* mutants and rescues. Control embryos, with wild-type Slit and Tok, have Slit-FL as well as both fragments. In the absence of *tok*, only Slit-FL is present. *tok* rescues have high levels of Slit-FL and specific Slit fragments. (B-F) Longitudinal axons visualized at late stage 17 with monoclonal antibody anti-Fasciclin 2 (Fas2). Anterior is upwards in all embryo images. Scale bars: 10 µm. (B) Control (*w^1118^*) embryos had three continuous longitudinal axon tracts running parallel to either side of the midline, totaling six longitudinal axon tracts, with no axons crossing the midline. (C) *tok^1^* homozygous embryos displayed disrupted longitudinal tracts with defasciculation (arrow) and missing outermost longitudinal fascicle (arrowhead). Axon tracts sometimes attempted to turn around (resulting in a J-shaped fascicle, not shown) or left the CNS entirely (asterisks; *n*=15). (D,E) Rescue of *tok* homozygous embryos with Slit-N, using *UAS-slit-N* under the control of the *single-minded-Gal4* (*sim*-*Gal4*) driver, had a range of phenotypes. (D) 53% of *tok^1^* embryos rescued with Slit-N fully restored longitudinal axons to wild-type numbers and morphology (arrowhead; *n*=8). (E) 40% of Slit-N rescues of *tok^1^* displayed an intermediate rescue phenotype, with significantly improved fasciculation and rescue of the outermost fascicle (arrowhead), but increased crossing of the innermost fascicle (arrow), not observed in *tok* mutants (*n*=6). (F) Expressing Slit-C in the midline, with *UAS-slit-C* and *sim-Gal4* in the absence of *tok^1^*, increased midline crossing at commissures (arrow) and slightly improved the number of longitudinal axons, but not fascicle organization. Axons still aberrantly left the CNS as in *tok* mutants (asterisks; *n*=11). (G) Quantification of the number of fascicles per segment for each genotype analyzed with a Kruskal–Wallis one-way ANOVA. Each point represents the number of fascicles at a single segment. Data are mean±95% CI. Differences from *w^1118^* are indicated over each bar and select pairwise comparisons of *tok* mutants to rescues are shown with horizontal bars (*P<0.05; ****P<0.0001). *tok* mutants are significantly different from wild type. The Slit-N rescue phenotypes were separated into two classes for clarity, but when pooled, the genotype is still statistically indistinguishable from wild type. See Fig. S2H for all pairwise comparisons.

Tok was implicated as the Slit protease in part because *tok* mutant longitudinal axon guidance defects are similar to those seen in the absence of Slit-N signaling (Alavi et al., 2016; Coleman et al., 2010; Serpe and O'Connor, 2006). The longitudinal axon defects of *tok* mutants could be due to the absence of Slit fragments, or to the persistence or increased amount of Slit-FL. Alternatively, the longitudinal axon defects could be caused by other proteins that Tok processes. To distinguish between these mechanisms, we performed genetic rescue experiments (Fig. 3A). Longitudinal axons are readily visualized with antibodies specific to Fasciclin 2 (Fas2), which reveals three axon fascicles on either side of the midline, totaling six fascicles in mature embryos (Fig. 3B). In *tok* mutants, longitudinal axons were greatly disrupted, failed to form distinct fascicles, stalled at segment boundaries and occasionally left the CNS entirely (Fig. 3C). However, midline repulsion was still largely intact, as the axons continued to avoid the midline. Avoidance of the midline is in contrast with *robo* and *slit* mutants, in which axons inappropriately enter the midline due to decreased midline repulsion (Seeger et al., 1993; Battye et al., 1999; Kidd et al., 1999). The *tok* phenotype supports previous evidence that Slit-FL alone is capable of midline repulsion, but not longitudinal axon guidance (Coleman et al., 2010). Additional *tok* alleles displayed similar phenotypes (Fig. S2B,C), and quantification of longitudinal defects revealed statistically significant differences compared with controls (Fig. 3G,Fig. S2G,H). We also evaluated a previously constructed uncleavable *slit* allele (Fig. S2D), and found that Slit-FL function may be disrupted by the addition of a C-terminal Myc tag (Fig. S2E), although it does not prevent Slit secretion or cleavage (Fig. S2F). These results strongly indicate that a Tok substrate, likely Slit, is required for longitudinal axon guidance.

Absence of Slit-Robo midline repulsion results in the complete collapse of all CNS axons onto the midline (Rothberg et al., 1990; Kidd et al., 1998), an attribute of Slit-FL (Coleman et al., 2010). Identification of the Slit protease allowed us to determine the functions of Slit fragments that were likely obstructed by the absence of Slit-FL in *slit* mutants. We expressed Slit-N and Slit-C in midline glia (*sim-Gal4*) in a *tok* mutant background. Expression of Slit-N at the midline dramatically rescued longitudinal fascicles as just over half of the embryos examined (53%) showed completely normal longitudinal axon fascicles, with no significant difference compared with wild-type controls (Fig. 3D,G). An additional 40% of Slit-N rescues showed an intermediate rescue of the three bilateral longitudinal fascicles, but also increased midline crossing of the innermost fascicles (Fig. 3E). Interestingly, a small number of Slit-N rescues (*n*=1, 7%) displayed a complete collapse of axons reminiscent of *slit* mutants (data not shown). There appears to be either attraction to the midline, aberrant fasciculation between innermost fascicles or interruption of Slit-FL repulsion when Slit-N is ectopically expressed. In contrast, expression of Slit-C caused an increase of midline crossing, but only at commissures, indicative of an issue at the segment boundary choice point as opposed to the pinching of innermost fascicles seen in intermediate Slit-N rescues (Fig. 3F). Although the number of longitudinal fascicles in Slit-C rescues is statistically improved compared with *tok* mutants, fascicles are still largely disorganized and in some places are indistinguishable from *tok* mutants (Fig. 3F,G). Our results suggest that Slit-N has a necessary biological activity for longitudinal axons that is distinct from axon repulsion, including as a positive mediator of longitudinal growth or fasciculation, and possibly attraction. The ability of Slit-N to rescue *tok* longitudinal phenotypes suggests that Slit is a major target of Tok in the CNS midline. Slit-C may have additional roles in axon guidance that were not detected by our analysis of longitudinal axons or were obscured by a relatively weak rescue and variable penetrance. Our results indicate that the CNS defects of *tok* mutants, namely reduced longitudinal axon guidance and defasciculation, are due to the absence of the Slit-N fragment, which may be promoting longitudinal axon growth or fasciculation, or both.

### Slit fragments cannot substitute for Slit-FL in midline repulsion

We previously observed that Slit-N expression has a weak effect on longitudinal axons in *slit* mutants when expressed by muscle precursors lying on top of the developing ventral nerve cord (Alavi et al., 2016). To further test whether either Slit-C or Slit-N could have independent repulsive functions, we expressed Slit-N and Slit-C in a *slit* mutant background using a pan-neuronal driver (*elav-Gal4*) to amplify any axon guidance phenotypes that may be obstructed by loss of Slit-FL. In the absence of Slit-FL, neither Slit-N nor Slit-C were able to rescue the collapsed axons (Fig. S3C,F). This was also true for Slit-N and Slit-C expressed under the control of a *single-minded Gal4* (*sim-Gal4*) driver (Fig. S3G-I). As expected, all CNS functions of Slit fragments were obscured by the complete collapse of CNS axons onto the midline in the absence of Slit-FL/Robo signaling. We confirmed *slit* transgene expression with antibody staining (Fig. S3C,F). As seen by other groups, both Slit-N and Slit-C antibody labeling appeared to localize to longitudinal axons in control embryos (Fig. S3A,D; Bhat, 2017). This localization of Slit to axons also occurred when expressing Slit-N, but not Slit-C, in rescues (Fig. S3C,F), indicating that Slit-FL and Slit-N tightly associate with the extracellular matrix, while Slit-C diffuses away (Brose et al., 1999).

We also evaluated differences in Slit-N and Slit-C localization in abdominal muscles and found that Slit-N, but not Slit-C, strongly localized to muscle attachment sites, consistent with data indicating a role for Slit-N, but not Slit-FL, in anchoring muscles to tendon cells (Fig. S4; Ordan and Volk, 2015). This is not surprising, as Tok has known functions in motor neurons, and *tok* mutants do not have the obvious muscle disruptions that *slit* mutants have (Meyer and Aberle, 2006; Serpe and O'Connor, 2006).

### Epistatic interactions between Tok and Slit *in vivo*

**Fig. 4.**
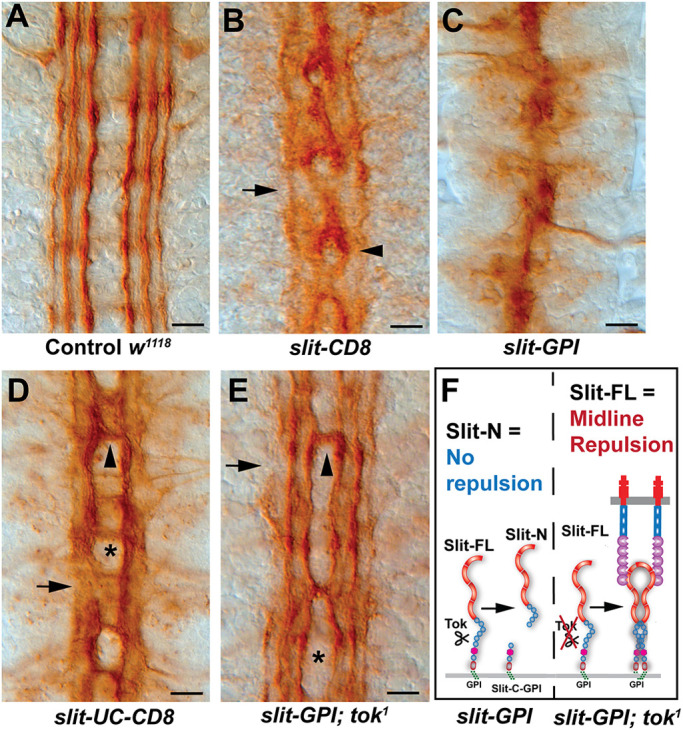
**Epistatic interactions between *tok* and membrane-tethered *slit*.** Stage 17 embryos labeled with anti-Fas2 for longitudinal axons. Anterior is upwards in all images. Scale bars: 10 µm. (A) Control embryos (*w^1118^*) have six distinct longitudinal fascicles with no fascicles crossing the midline. Midline repulsion ensures that bilateral axons run contralateral to the midline, separated by midline glia. (B) A recombinant *slit* allele that is wild type, except for the addition of a CD8 transmembrane anchor to the C terminus (*slit-CD8*), showed that the two innermost axon tracts of each side fasciculated to form a single roundabout on the midline (arrowhead), but maintained an outermost fascicle (arrow; *n*=9). (C) Embryos homozygous for a *slit* allele in which a GPI anchor tethers Slit to the membrane (*slit-GPI*) showed the complete collapse of CNS axons onto the midline (*n*=9). (D) A recombinant *slit* allele with both a CD8 transmembrane anchor on the C terminus and lacking the Slit cleavage site (*slit-UC-CD8*) had somewhat normal midline repulsion (asterisk), with some instances of midline crossing at commissural sites (arrowhead) and intermittently defasciculated outermost axon tracts (arrow; *n*=10). (E) A double mutant of *slit-GPI* and *tok^1^* generally rescued midline repulsion compared with *slit-GPI* alone (asterisk) but failed to rescue longitudinal axon defects (arrow) and had fascicles that occasionally crossed the midline (arrowhead; *n*=9). (F) Models of the Slit-GPI and Tok protein interaction. Slit-GPI is constitutively cleaved by Tok to produce Slit-N and membrane-tethered Slit-C. As the Slit-N fragment lacks repulsive activity, this cleavage leads to the collapse of CNS axons onto the midline in Slit-FL-GPI embryos. Removing Tok-mediated Slit cleavage restores the Slit-FL-GPI midline repulsive activity, by making Slit-FL available to Robo receptors on CNS axons.

Alleles encoding membrane-tethered versions of diffusible ligands have been informative in dissecting short- and long-range effects of axon guidance cues (Brankatschk and Dickson, 2006; Park et al., 2011). Slit-N closely associates with CNS axons, indicating they may have a short-range function in longitudinal fasciculation and growth (Fig. S3A,C). To test the short-range functions of Slit, we analyzed *slit* alleles that artificially tether Slit to the cell membrane, created by homologous recombination at the endogenous *slit* locus. Membrane-tethered alleles of *slit* have been analyzed in muscle formation, but not in the CNS (Ordan et al., 2015). Two distinct anchors were used to tether Slit to the plasma membrane, a CD8 transmembrane domain and a GPI anchor (Fig. S5A). Labeling of a *slit-CD8* allele with Fas2 antibody showed a strong resemblance to *robo* mutants, as the outermost 1D4-positive fascicle was largely intact, but the innermost and medial bundles fasciculated and repeatedly crossed the midline, forming axon circles resembling roundabouts (Fig. 4B). This suggests that the altered Slit protein retains some repulsive activity and that the fascicles most distant from the Slit source can sample the tethered Slit effectively. In early embryos, filopodia can extend across the midline (Murray and Whitington, 1999), and the elaborate axonal arbors of multidendritic neurons suggests that growth cones can explore all areas of the neuropil in older embryos (Grueber et al., 2007). In contrast, the *slit*-*GPI* allele appeared to lack all repulsive activity as it resembled a *slit* loss-of-function allele (Fig. 4C). This suggested that Slit-GPI is completely inactive. Interestingly, an uncleavable variation of *slit-CD8* (*slit-UC-CD8*) qualitatively reduced the midline crossing phenotype compared with *slit-CD8* alone as the stereotypical ‘roundabouts’ of the *slit-CD8* mutant are no longer present (Fig. 4D). The phenotypic improvement in repulsion of uncleaved anchored Slit compared with cleavable *slit-CD8*, combined with our finding that Slit-N did not mediate repulsion (Fig. S3), suggests that membrane tethering may lead to more efficient proteolytic processing of the Slit-CD8 protein. Provided the Slit-CD8 protein is proteolytically cleaved more than wild-type Slit, the increased release of Slit-N and reduction of Slit-FL will result in reduced repulsion in *slit*-*CD8* mutants, which will be rescued in *slit-UC-CD8*.

The rescue of repulsion with an uncleavable, anchored allele also suggested that the *slit-GPI* allele might be even more efficiently processed by Tok, resulting in no repulsive Slit-FL protein, based on the severity of the collapse phenotype. To test this, we constructed a *slit-GPI; tok^1^* double mutant and found that midline repulsion was dramatically restored compared with *slit-GPI* alone (Fig. 4E;Fig. S5B). The simple model that *slit-GPI* is non-functional was eliminated by the restoration of repulsive function in the *slit-GPI; tok^1^* double mutant. When protease activity is absent, Slit-GPI acts as a repulsion cue for axons at the midline. We favor the explanation that membrane tethering enhances Slit processing, similar to receptor binding (Ordan and Volk, 2015). In *slit*-*GPI* mutants, Tok constitutively cleaved Slit-FL-GPI and released Slit-N, while leaving behind an anchored Slit-C-GPI. This resulted in increased Slit-N compared with Slit-FL, as visualized by immunoblot (Fig. S5C). As neither Slit-N nor Slit-C acted as midline repellents (Fig. S3), and Slit-FL is not available, all axons collapse onto the midline (Fig. 4C,F; Fig. S5B). Preventing Tok cleavage resulted in Slit-FL-GPI remaining on the midline glia and repelling axons. As the removal of *tok* results in no Slit-N (Fig. 1B), *slit-GPI; tok* double mutants still have some longitudinal axon defects, similar to the membrane-tethered uncleavable Slit (Fig. 4D;Fig. S5B). However, we did note that the longitudinal axon defects are improved from *tok* mutants alone. Although we do not know the exact mechanism for this, we speculate that the circulating uncleaved Slit-FL in *tok* mutants could contribute to axon disruption, which is reduced when Slit is tethered. Allele-specific epistatic interactions are often indicative of physical interactions (Domingo et al., 2019), and the *slit-GPI; tok* phenotype supports our data that Tok is the Slit protease and further emphasizes that Slit-N is distinct from Slit-FL *in vivo*. It also highlights that dissecting the short- and long-range activities of Slit may be more challenging than for other ligands.

### *tok* phenotypes arise from stalling defects in pioneer longitudinal axons

**Fig. 5.**
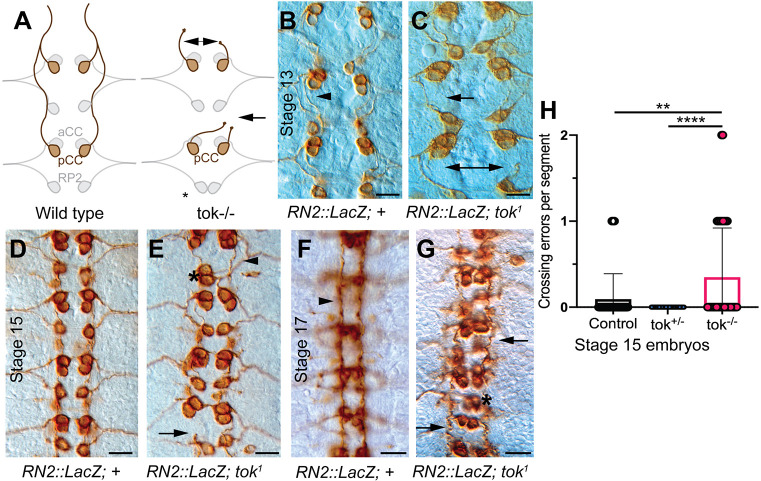
**Labeling of pioneer axons showed early defects at the segment boundary in *tok* mutants.** (A) Schematic of RN2::LacZ labeled neurons in wild type and *tok* mutants, showed stalling of the posterior corner cell (pCC) axons and inappropriate crossing of the CNS midline in *tok* mutants. (B-G) Visualization of pioneer axons using an RN2-Gal4:UAS-τ-LacZ reporter construct, which expresses LacZ in pioneer axons, labeled with β-gal, was used to assess single-axon guidance in stage 13 (B,C), stage 15 (D,E) and stage 17 (F,G) embryos. Anterior is upwards in all images. Scale bars: 10 µm. (B) Stage 13 control embryo (RN2::τLacZ;+) showed normal pCC axons crossing the segment boundary (arrowhead) at similar growth rates on either side of the midline. (C) Stage 13 homozygous *tok^1^* mutants showed missing pCC axons and axons growing at different rates across the segment boundary (arrows; *n*=7). (D) In stage 15 control embryos, pCC axons extended anteriorly in all segments, with evenly spaced cell bodies. (E) In stage 15 embryos homozygous for *tok^1^*, neuronal cell bodies were generally misplaced, most especially RP2 (asterisk). Axons grew at variable rates or stalled while crossing segment boundaries (arrow), but some pCC axons crossed normally (arrowhead) (*n*=5). pCC axons occasionally crossed the midline at the closest anterior commissure (not shown). (F) Control stage 17 embryos showed stereotypical straight, condensed axon tracts (arrowhead). (G) *tok^1^* stage 17 embryos had poorly condensed, vermicular pCC axons (arrows) and misplaced cell bodies (asterisk; *n*=8). (H) Quantification of crossing errors per segment, defined as stalling or prematurely crossing the midline, were performed for stage 15 embryos (Kruskal–Wallis one-way ANOVA, **P<0.01; ****P<0.0001). Data are mean±95% CI.

To analyze the origins of the *tok* longitudinal axon phenotype, we observed longitudinal axons using an RN2 construct driving lacZ. This construct allowed us to visualize pioneer axons, which are the first axons to cross the segment boundary, laying a framework for subsequent axons to fasciculate and follow (Fig. 5A). *tok* mutant pioneer axons stalled during the initial projection of axons across the segment boundary (stage 13) and sometimes prematurely crossed the midline or exited the CNS (Fig. 5C,E,H). *tok* mutants also showed generally disorganized neuron cell bodies, but no reduction in the number of neurons, indicating normal cell fate decisions, but less organization around the midline. Lack of Slit-N in *tok* mutants, therefore, likely reduces the ability of pioneer axons to cross the segment boundary. This result suggests that Slit-N directly stimulates pioneer axon outgrowth to push extending axons over the segment boundary. Disruption to the early axon scaffold was amplified in later stages as subsequent axons compound the guidance errors (Fig. 5E,G).

## DISCUSSION

Slit is a canonical CNS midline repellent, but with the potential to generate diverse guidance signals via proteolytic processing. Numerous studies have acted on the assumption that Slit-N is the only biologically active Slit isoform in neural development, principally owing to the presence of the Robo-binding site. However, the work presented in this article demonstrates that Slit-FL and Slit-N have dramatically different activities *in vivo*. The key to these findings was the identification of Tok as the Slit protease, allowing *in vivo* genetic manipulation of Slit fragments to uncover the independent biological function of Slit-N. Our results complement the few studies in which Slit-FL and Slit-N have been observed to have distinct activities (Gohrig et al., 2014; Nguyen Ba-Charvet et al., 2001; Wang et al., 1999) as well as those that have attributed activities to a specific Slit fragment (Wright et al., 2012; Ducuing et al., 2020 preprint; Caipo et al., 2020). As Slit cleavage and Tok family proteases are evolutionarily conserved (Brose et al., 1999; Hopkins et al., 2007), it seems highly likely that processing of Slits by BMP-1/Tolloid family proteases will diversify the biological output of Slit signaling in many different systems.

### Proteolytic cleavage diversifies Slit signaling in axon guidance

**Fig. 6.**
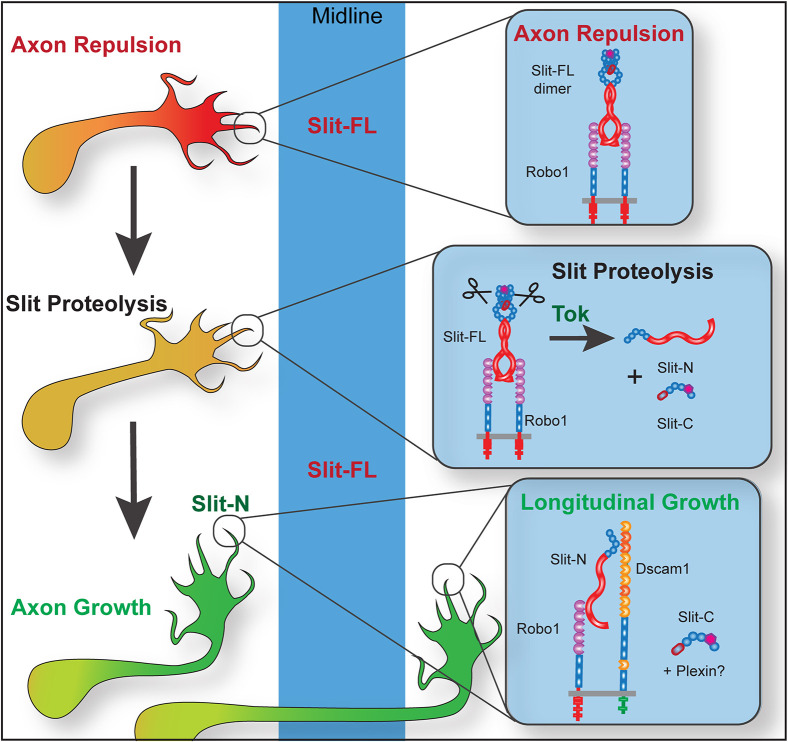
**Model for receptor-mediated cleavage of Slit and functions for individual Slit fragments.** In this model of midline CNS axon guidance, axons approach midline sources of full-length Slit and are repelled through Slit-FL/Robo signaling. Upon Robo receptor binding, Slit-FL is available for Tok-mediated Slit proteolysis to generate Slit-N and Slit-C fragments. Slit-N is then able to form a tertiary complex with Robo1 and Dscam1 to promote longitudinal axon growth. Although we do not identify a specific function for Slit-C, it may be capable of binding plexins to elicit other CNS responses (Delloye-Bourgeois et al., 2015).

In the context of axon guidance *in vivo*, why is Slit cleaved? There are remarkably few identified ligands for axon guidance, and the ability to diversify a single signal by proteolysis increases the information available to navigating axons. The same cue could theoretically promote axon attraction, repulsion, growth and fasciculation, depending on the proteolytic state. In the *Drosophila* CNS midline, we favor a model in which Slit-FL repulsion is balanced with Slit-N-mediated axon growth regulated by Tok proteolytic activity. In a sequence of events, Slit-FL would promote midline repulsion by binding to Robo receptors, and receptor binding would then allow Tok to process some, but not all, Slit. Subsequently, the newly produced Slit-N can recruit specific co-receptors such as Dscam1 to promote longitudinal axon growth (see Fig. 6). Dscam binds EGF domains 1-3 of Slit (Alavi et al., 2016), which are apparently inaccessible until after proteolysis. Deletion of the EGF domains converts Slit2-N from a chemoattractant into a chemorepellent for human neutrophils (Pilling et al., 2019). Expression of a Slit transgene lacking EGF domains 2-6 decreased midline crossing and disrupted longitudinal axon guidance in flies (Battye et al., 2001), consistent with increased levels of Slit-FL. The balance between Slit-FL and fragment levels is therefore likely to be important, and regulation of Slit proteolysis will be crucial.

### Slit-N in longitudinal axon guidance and fasciculation

What is the specific function of Slit-N in longitudinal axon guidance? The longitudinal and pioneer axon guidance phenotypes are seen in mutants lacking Slit-N and rescued by expression of Slit-N (Figs 3 and 5) support a model of Slit-N acting as a positive factor. Growth cones require stimulation of axon growth to cross the segment boundary (Kuzina et al., 2011), and our data suggest that Slit-N is one of the factors promoting this growth. Attractive and repulsive cues have been suggested to synergistically stimulate longitudinal axon growth parallel to the midline in vertebrates, in a ‘push-pull’ model (Kim et al., 2014). In some cases, Slit-N may even attract axons to the CNS midline (Fig. 3E), suggesting that processing Slit into an attractive cue might generate a similar synergy.

Slit-N may also promote axon fasciculation. Prior observations have suggested that Slit-N tightly binds the matrix, whereas Slit-C is diffusible (Brose et al., 1999; Svensson et al., 2016). Matrix associated Slit-FL/Slit-N has been proposed to maintain longitudinal axon fasciculation (Bhat, 2017). Slit-N may bridge Robo receptors on muscle and tendon cells (Ordan and Volk, 2015), and Slit/Robo signaling is required for the clustering of pulmonary neuroendocrine cells (Branchfield et al., 2016). Our observations of Slit-N axonal localization suggest it could promote axon fasciculation, as demonstrated for Slit2-N in spinal motor axons (Jaworski and Tessier-Lavigne, 2012), which would contribute to the recovery of organized longitudinal fascicles in our rescues. The persistence of Robo protein localization on longitudinal axons also suggests Slit-N could be playing a structural role in maintaining fasciculation (Kidd et al., 1998). Fasciculation could render the axons less sensitive to midline Slit-FL and promote axon growth (Bak and Fraser, 2003). Additionally, Slit-N could mediate axon-glia interactions required for longitudinal axon guidance or could be overriding Slit-FL repulsion with adhesion (Hidalgo and Booth, 2000).

### Slit-C and alternate receptors

What function does Slit-C play? Although we did not find substantial evidence of Slit-C function at the CNS midline, it is possible that Slit-C may be binding Plexin receptors and transducing a repulsive signal as observed in vertebrates (Delloye-Bourgeois et al., 2015). *Plexin* mutations in the fly produce longitudinal axon disruptions (Wu et al., 2011) and PlexinB has a distinct role in axon bundling of longitudinal axons (Ayoob et al., 2006). Slit-C could also be mediating axon-matrix adhesive interactions via dystroglycan binding, which interacts at the C-terminus of Slit2 in vertebrates, although whether dystroglycan binds just Slit2-C or also Slit2-FL is untested (Wright et al., 2012). Our results are distinct from findings that Robo2 in flies and Robo3 in vertebrates can act as inhibitory receptors to promote axon attraction (Englund et al., 2002; Evans et al., 2015; Sabatier et al., 2004), although it is likely that there will be additional receptors for both Slit-N and Slit-C, and Slit-N binding to Robo2 and Robo3 has not been tested. Other Dscam family members are likely to bind Slit-N in neurons but Slit-N also has effects on the vasculature, where Dscam expression has not been reported (London and Li, 2011). Additional Slit-FL, Slit-N and/or Slit-C receptors may explain disparities in Slit outputs seen in cultured neuron assays. For example, Slit2-FL and Slit2-N both repel olfactory bulb axons but have antagonistic effects on the growth and branching of DRG axons (Nguyen Ba-Charvet et al., 2001).

### Regulating Slit processing

Understanding how Slit proteolysis is regulated *in vivo* will be a crucial next step in understanding Slit/Robo signaling. Analysis of Slit cleavage in *Drosophila* embryos reveals a balance between Slit-FL and Slit fragments, with most Slit remaining in the full-length form during embryogenesis (Furrer et al., 2007), and Slit-FL is required to conduct midline repulsion (Coleman et al., 2010). Our data also suggest that Tok cleavage of Slit is tightly regulated because overexpressing Tok *in vivo* does not generate gain-of-function phenotypes, whereas the same constructs rescue *tok* mutants (Serpe and O'Connor, 2006). This suggests that the activity of Tok is regulated at both transcriptional and post-transcriptional levels, such as the cleavage of a pro-sequence required to activate Tok (Hopkins et al., 2007). Slit cleavage likely only occurs in certain contexts, such as when associated with the cell membrane by receptor or matrix binding (Ordan and Volk, 2015). Structure-function analysis of both Tok and Slit, both highly modular proteins, may shed light on how regulation of Slit fragment signaling occurs.

There are clear structural and functional overlaps between members of the Tolloid protease family (Hopkins et al., 2007; Serpe and O'Connor, 2006). In flies, Slit cleavage occurs in the early embryo at a time when Tolloid expression is high, and Tok is not known to be expressed, suggesting Tolloid can cleave Slit. Nevertheless, fly *tolloid* (*tld*) expression cannot rescue *tok* motor neuron defects, and *tok* cannot rescue *tld* (Nguyen et al., 1994; Serpe et al., 2005). The specificity of Slit proteolysis may rely primarily on differences in expression patterns of the proteases, and the kinetics of substrate processing. As such, other members of the BMP-1/Tolloid family should be investigated as potential, if likely inefficient, Slit proteases.

In vertebrates, Tll1 and Tll2 appear to be Tok homologs (Clark et al., 1999), although functional overlaps and differences with BMP-1/Tolloid family members have been observed (Pappano et al., 2003; Zhang et al., 2017). We expect that mutants for Tll1 or Tll2 in vertebrates may display a subset of Slit-mediated axon guidance phenotypes, revealing Slit fragment functions. Finally, numerous assay systems have been used to examine the effects of Slit/Robo signaling in different biological contexts, ranging from organogenesis to stem cell regulation (Blockus and Chédotal, 2016). Functional differences between Slit-FL and Slit-N are rarely tested, but antagonism has been observed in a neural invasion and metastasis model of pancreatic cancer (Gohrig et al., 2014). This suggests that the many and diverse Slit functions should be revisited, particularly those examining Slit protein as a potential biotherapeutic agent.

## MATERIALS AND METHODS

### Fly lines

Standard genetic techniques were used to create double mutant and balanced stocks of *Drosophila melanogaster*. Recombinant Slit alleles (*slit-Myc*, *slit-UC-Myc*, *slit-CD8*, *slit-UC-CD8* and *Slit-GPI-Myc*) were a gift from the Volk lab (Weizmann Institute of Science, Rehovot, Israel) and produced in the Dickson lab (Janelia Research Campus, Ashburn, VA, USA). The alternate *tok* allele *tlr^X2-41^* was from M. O'Connor (University of Minnesota, USA). *UAS-slit-N* and *UAS-slit-C* lines were obtained from G. Bashaw (University of Pennsylvania, USA), Volk Lab and R. Jacobs (McMaster University, Ontario, Canada). All other fly lines came from Bloomington Stock Center, including *tok^1^, tok^3^, tok^MI06118-TG4.1^* (76679), *tld^2^, Df(3R)^BSC519^* (25023), *slit^2^*, *w^1118^*, *amon^C241Y^*, *amon^Q178^st*, *RN2-Gal4:UASτ-LacZ*, *10XUAS-CD8::GFP* (32184) and *sim-Gal4*. Stocks were balanced over *CyO GMR-YFP, CyO weeP* or *TM6B GMR-YFP* for protein extraction. Protein extraction was conducted with stage 16 to early larval 1 embryos. All flies were maintained at 25°C except for *slit* rescue experiments, which were carried out at 29.5°C (Fig. S3). Examination of the axon phenotypes and Slit cleavage in homozygous embryos led to the identification of a *commissureless* mutation in the *tok^3^* background, and the conclusion that only *tok^1^* is a true null allele.

### Cell lines

*Drosophila* S2 cells were grown in Schneider's *Drosophila* Medium (Thermo Fisher Scientific) with 10% FBS (Atlanta Biologicals) at 28°C. COS-7 cells were grown in DMEM with 10% FBS at 37°C in 5% CO_2_. *Drosophila* S2 cells and COS-7 cells were gifts from other UNR labs, as detailed in Alavi et al. (2016). Cell lines were not authenticated or tested for contamination.

### Slit cleavage analysis of whole-embryo lysates

Whole-embryo protein lysates were collected using 100 staged (stages 16-early larval 1) embryos homogenized in 50 µl cell lysis buffer [50 mM HEPES (pH 7.2), 100 mM NaCl, 1 mM MgCl_2_, 1 mM CaCl_2_ and 1% NP-40) with protease inhibitor cocktail (Sigma). Embryos were then centrifuged and analyzed using SDS-PAGE (4-20% gradient) and immunoblot with anti-Slit-C antibody (1:100 dilution, DSHB C555.6D; Rothberg et al., 1988), anti-GAPDH (1:1000 dilution, Millipore Sigma AB2302) and anti-Slit-N [1:1000, monoclonal antibody generated by Bland (2001). Western blots were imaged using a ChemiDoc Touch Imaging System. Following antibody labeling with anti-Slit-C, some blots were stripped with a stripping buffer (10% SDS, 0.5 M Tris-HCl and β-mercaptoethanol) for 1 h at 37°C and reprobed with the anti-Slit-N antibody.

The C555.6D antibody was created using a bacterially expressed TrpE fusion protein consisting of the laminin G/ALPS domain and the 7th EGF repeat as an immunogen (Rothberg et al., 1988). A non-specific 75 kDa band is frequently observed on immunoblots when using the C555.6D anti-Slit-C monoclonal (Fig. 1B; Bhat, 2017). The band is present in extracts of *slit^2^* null mutants (Fig. 1B), indicating that it is non-specific. The band can give a remarkably strong signal (see Fig. 8A in Bhat, 2017) with the potential to be mistaken for the 50-55 kDa Slit-C fragment if an immunoblot is cut above the 55 kDa marker band. For example, the commonly run loading control alpha-Tubulin runs at 55-60 kDa, leading to potential loss of the Slit-C band unless the blot is re-probed or an alternate loading control is used. Additional non-specific bands can also be observed, such as a 120 kDa band (Furrer et al., 2007). We have also observed bands above 200 kDa in *slit* mutant extracts. The Slit-C fragment is sometimes not detectable in embryo extracts (Bhat, 2017), so independent replicates are essential. The anti-Slit-N monoclonal antibody 10B2 used in this paper was created in the Goodman laboratory by K. Bland using EGF repeats 1-3 expressed as a 6xHIS bacterial fusion protein as an immunogen (Bland, 2001). Antibody is no longer available as the monoclonal cell line was accidentally lost.

### Slit cleavage analysis of *in vitro* media lysates

*Drosophila* S2 cells were grown in Schneider's *Drosophila* Medium (Thermo Fisher Scientific) with 10% FBS (Atlanta Biologicals). The pRmHaIM-Tok (Serpe et al., 2005) and pIB-Slit (Alavi et al., 2016) plasmids were co-transfected using the Cellfectin II reagent (Thermo Fisher Scientific) according to the manufacturer's instructions. Seventy-two hours post-transfection, cell medium was collected and analyzed via western blot. The full-length Slit and Slit-C cleavage products were detected with Slit-C antibody at a 1:50 dilution (anti-C555.6D, DSHB) and Slit-N antibody at 1:500 (courtesy of K. Bland, Stowers Institute for Medical Research, Kansas City, MO, USA).

Transfections with DNA expression constructs were performed on COS-7 cells in a six-well plate at 80% confluence using Lipofectamine 2000 or 3000 (Life Technologies), according to the manufacturer's instructions. pcDNA3.1^+^/C-DYK-Drosophila-Slit-FL (Alavi et al., 2016) and pcDNA3.1^+^/C-DYK-Drosophila-Tok (synthesized by GenScript) were transfected into COS-7 cells. Media were replaced with fresh DMEM (Gibco) containing 2% FBS 24 h post-transfection. At 48 h post-transfection supernatant was harvested and the cells were released using cell lysis buffer and homogenized. Cell lysates and supernatant were analyzed with SDS-PAGE and the blot was probed with a primary anti-Myc antibody (mouse monoclonal, 1:500, Abcam, ab32).

### Embryo immunohistochemistry

*Drosophila* were reared at 25°C except for *slit^2^* rescue experiments, which were incubated at 29.5°C. Whole-embryo *Drosophila* labels were performed as described previously (Patel, 1994). Anti-Fas2 (1:5, 1D4) and Slit-C (1:10, C555.6D) monoclonal antibodies were obtained from the Developmental Studies Hybridoma Bank (DSHB). Anti-Slit-N (1:100) is a monoclonal antibody generated by K. Bland (Bland, 2001). Anti-β-Galactosidase (1:10,000, 0855976) was obtained from MP Biomedicals and anti-GFP (1:250, A11122) was obtained from Thermo Fisher Scientific. Staining Slit-N and Slit-C with NiCl_2_ and anti- β-Gal RN2::LacZ was enhanced with Vectastain ABC (Vector Labs). HRP-conjugated secondary antibodies (1:500, 115-035-003, 111-035-003 and 111-065-003) and fluorescent secondary antibodies (1:250, 711-545-152, 200-002-211 and 016-600-084) were obtained from Jackson ImmunoResearch. Dissected and stained embryos were imaged on a Leica DM5000B with a Jenoptix ProgRes C5, using the ProgRes Mac CapturePro software. Fluorescently labeled embryos were dissected and imaged on a Leica CTR5500 with a Leica DFC350FX camera and a Leica Thunder Imager 3D Tissue Model DM6B with a Leica DFC9000GT camera. *Z*-stack movie is a compilation of 134 images taken over 211.2 µm. Fluorescent images underwent Thunder computational clearing. The movie was edited using TechSmith Camtasia 2019.0.10.

### Quantification and statistical analysis

#### Quantification of Slit cleavage

##### Whole-embryo lysates

Analysis of Slit cleavage was carried out using ImageJ, by comparing relative pixels of Slit-C to Slit-FL, after standardizing to GAPDH loading controls. Slit-C:Slit-FL ratios were averaged over all blots, consisting of at least two independent embryo collections for each genotype and at least three total blots. *slit^2^* was excluded from quantification because significantly reduced Slit protein conflates the Slit-C:Slit-FL ratio. Differences between averaged Slit-C:Slit-FL ratios were compared with a one-way Welch ANOVA with Dunnett's T3 multiple comparisons test with GraphPad Prism 8.4.3.

##### COS-7 cells

Blots were analyzed using ImageJ to generate relative ratios of protein expression using the pixels in each band. Percentage of Slit-FL was compared between the pcDNA3.1^+^/C-DYK-Drosophila-Slit-FL transfected condition and the pcDNA3.1^+^/C-DYK-Drosophila-Slit-FL and pcDNA3.1^+^/C-DYK-Drosophila-Tok co-transfection conditions. The relative pixels of Slit-C and Slit-FL were analyzed via ImageJ. A Welch two-sample *t*-test was used to determine the statistical significance of the reduction of FL-Slit in the presence of Tok (*n*=9).

#### Phenotypic quantification

##### Longitudinal axons

Quantification of longitudinal axon defects were characterized using stage 17 embryos or early stage 1 larvae. Number of anti-Fas2 labeled fasciculated tracts were blindly counted at the segment boundary for abdominal and thoracic segments for each embryo, with at least eight segments scored. Number of fascicles per segment were averaged for each genotype and the data were analyzed with a Kruskal–Wallis one-way ANOVA with Dunnett's T3 multiple comparisons test with GraphPad Prism 8.4.3.

##### pCC axon errors

pCC axon crossing errors were blindly quantified using stage 15 embryos, as embryos at stage 13 still have some migrating axons and condensation of the nerve cord by stage 17 makes the segment boundary difficult to see. The number of pCC axon errors at each segment boundary was blindly counted. Normally crossing bilateral axons were scored at zero. Axons that failed to cross the segment boundary or crossed at the immediate posterior segment were given a score of one, with the maximum number of errors per segment being 2. Number of axon crossing errors per segment were averaged for each genotype and analyzed using a Kruskal–Wallis one-way ANOVA with Dunnett's T3 multiple comparisons test with GraphPad Prism 8.4.3.

## Supplementary Material

Supplementary information
