# Supplementary material for: Proteolytic cleavage of Slit by the Tolkin protease converts an axon repulsion cue to an axon growth cue in vivo
Source: Development. 2020 Oct 29;147(20):dev196055. doi: 10.1242/dev.196055 (PMC7648596; doi:10.1242/dev.196055)
Supplement: Supplementary information [file develop-147-196055-s1.pdf]

Supplementary Material

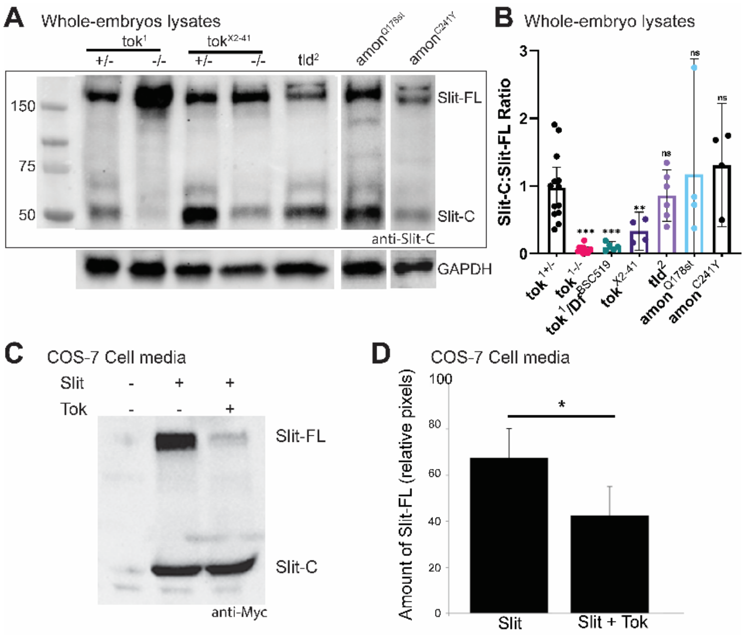

Figure S1. Additional evidence that Tok is the Slit protease. (A) Immunoblot analysis of additional *tok* alleles *in vivo* with whole-embryo lysates and antibody specific to the C-terminus of Slit (C555.6D). Slit cleavage was reduced in a *tok* hypomorph, *tok*$^{X2\text{-}41}$, but maintained in *tolloid* (*tld*^{2}) mutants and homozygous mutants of two *amontillado* alleles (*amon*^{Q178st} and *amon*^{C241Y}). (B**)** The intensity of the Slit-FL and Slit-C bands of *tok* and other candidate genes on western blots was quantified and expressed as a ratio of Slit-C to Slit-FL to analyze the amount of cleavage. Slit processing was significantly reduced in *tok*^{1} homozygotes, *tok*$^{1/}$*Df(3R)*^{BSC519} embryos, and *tok*$^{X2\text{-}41}$ hypomorphs (** p=0.0096; n=3 independent experiments). Cleavage was unaffected in *tld*^{2} (n=5 independent experiments), *amon*^{Q187st} (n=3 independent experiments), and *amon*^{C241Y} (n=3 independent experiments) homozygotes. Differences in cleavage were analyzed with a Welch one-way ANOVA (*tok*$^{1\,+/-}$; *** p<0.001). Data are represented as mean ± 95% CI. **(C)** When transfected with Slit-FL, COS-7 cells endogenously cleave Slit in the media. When co-transfected with plasmids for Slit-FL with a C-terminal -Myc tag and Tok, the amount of Slit cleavage increased, visualized as a decrease in levels of Slit-FL in media. Slit-FL and Slit-C were probed with anti-Myc antibody. **(D)** Quantification of the amount of full-length Slit in COS-7 cell transfections with or without Tok showed a significant reduction in Slit-FL (Welch 2 sample t-test, * p=0.048; n=4 independent experiment).

Development • Supplementary information

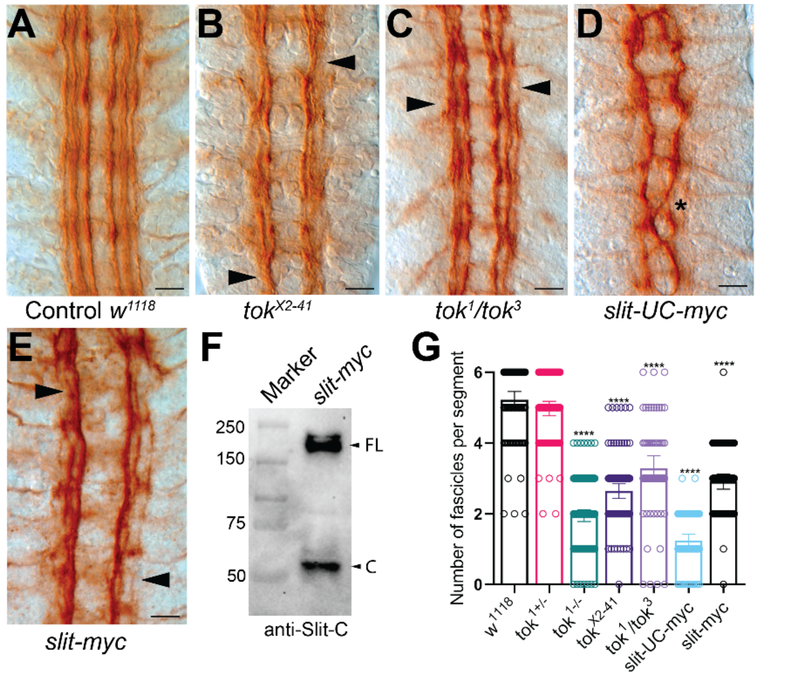

H

Genotype	w^{1118}	$tok^{1+/-}$	$tok^{1-/-}$	sim:slit-C; $tok^{1-/-}$	sim:slit-N; $tok^{1-/-}$(Full)	sim:slit-N; $tok^{1-/-}$(Int.)	tok^{X2-41}	tok^1/tok^3	slit-UC-myc	slit-myc
w^{1118}	-	>0.9999	<0.0001	<0.0001	>0.9999	0.0091	<0.0001	<0.0001	<0.0001	<0.0001
$tok^{1+/-}$		-	<0.0001	<0.0001	0.6197	0.0859	<0.0001	<0.0001	<0.0001	<0.0001
$tok^{1-/-}$			-	<0.0001	<0.0001	<0.0001	0.0818	<0.0001	0.2513	0.0007
sim:slit-C; $tok^{1-/-}$				-	<0.0001	>0.9999	0.0761	>0.9999	<0.0001	>0.9999
sim:slit-N; $tok^{1-/-}$(Full)					-	<0.0001	<0.0001	<0.0001	<0.0001	<0.0001
sim:slit-N; $tok^{1-/-}$(Int.)						-	0.0001	>0.9999	<0.0001	0.0235
tok^{X2-41}							-	0.8111	<0.0001	>0.9999
tok^1/tok^3								-	<0.0001	>0.9999
slit-UC-myc									-	<0.0001
slit-myc										-

Figure S2. Longitudinal axon guidance defects in *tok* and *slit* mutants. (A-E) Embryos labeled with anti-Fas2 to show longitudinal CNS axons. Embryos are stage 17 and anterior is up in all images, bar indicates 10µm. (A) w^{1118} control embryo. (B, C) Additional *tok* mutants, such as tok^{X2-41} homozygotes (n=11) or transheterozygous tok^1/tok^3 embryos (n=7), had longitudinal breaks or defasciculation in the outermost fascicles (arrowheads). (D) Recombinant uncleavable *slit* allele (*slit-UC-myc*) with a C-terminal -Myc tag showed a *robo*-like phenotype with longitudinal tracts collapsed into two fascicles that repeatedly cross the midline (asterisk; n=9). This allele was phenotypically distinct from *tok* mutants, leading us to evaluate the role of the -Myc tag in longitudinal axon guidance. (E) A recombinant *slit* allele with a C-terminal -Myc tag (*slit-myc*) that is otherwise wild-type in amino acid sequence showed strong longitudinal axon phenotypes with thin and absent outermost fascicles (arrowheads; n=9). (F) Western analysis of *slit-myc* homozygous whole-embryo lysates probed with anti-Slit-C to confirm expression and cleavage of Slit with Slit-FL (~180 kDa), and Slit-C (~55 kDa) present (n=3). (G) Quantification of

Development • Supplementary information

longitudinal axon defects. For each genotype shown, the number of fascicles at each segment boundary were counted in embryos labeled with anti-Fas2 and averaged by genotype. *w*^{1118} (n=10) was used as a wild type control, which averages 5-6 fascicles per segment. Each point represents the number of fascicles in a single segment. Genotypes were compared using a Kruskal-Wallis one-way ANOVA. The statistical difference from *w*^{1118} is indicated over each bar (**** p<0.001). Data represent mean ± 95% CI. (H) Table of p-values for all genotypic comparisons of the number of fascicles per segment from Fig. 3 and Fig. S2.

Development • Supplementary information

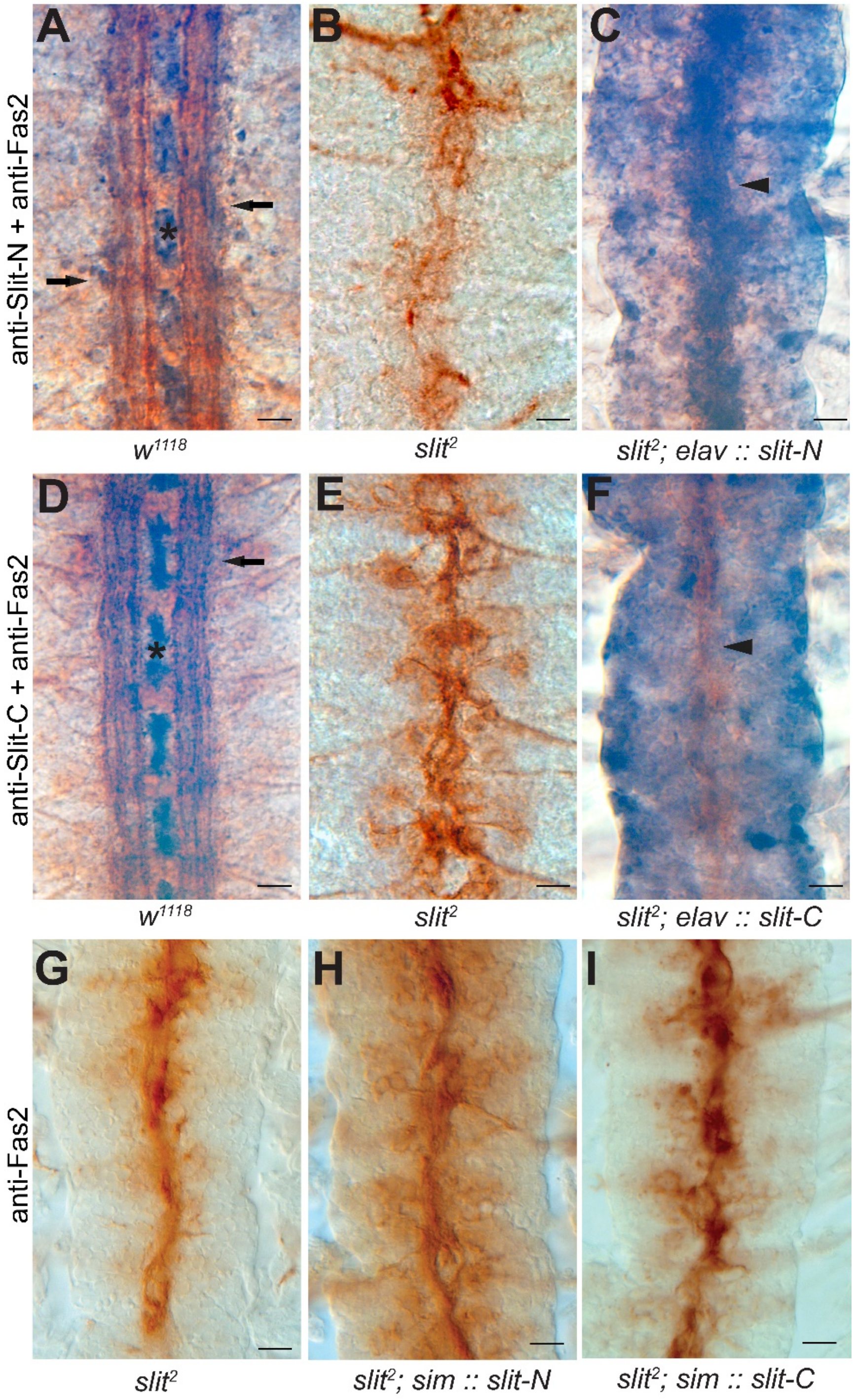

Figure S3. Slit fragments are unable to rescue *slit* mutants. Embryos labeled with anti-Fas2 (brown) and anti-Slit-N (purple; A-C) or anti-Slit-C (purple; D-F) to assess the ability of Slit fragments to rescue *slit* mutants. All embryos were treated identically and are stage 17. Anterior is up in all images and bar indicates 10µm. (A) w^{1118} control embryos labeled with Slit-N antibody showed the distribution of Slit in midline glia (asterisk), with Slit-N labeling also visible along the outermost longitudinal fascicle (arrows;

Development • Supplementary information

n=3). (B) *slit*^{2} homozygous mutants had a characteristic collapse of CNS axons onto the midline, with no apparent Slit-N antibody labeling visible (n=4). (C) Expressing Slit-N with a pan-neuronal driver (*elav-Gal4*) and *UAS-slit-N* in a *slit*^{2} mutant background did not rescue midline repulsion of the CNS axons, though Slit-N still localized to axons (arrowhead; n=3). (D) *w*^{1118} control embryos labeled with Fas2 and Slit-C antibody showed expression in midline glia (asterisk) and some distribution to longitudinal fascicles (arrow; n=4). (E) *slit*^{2} homozygous mutants had a characteristic collapse of CNS axons onto the midline, with no apparent Slit-C antibody labeling visible (n=3). (F) Expressing *UAS-slit-C* with *elav-Gal4* in a *slit*^{2} mutant background did not rescue midline repulsion, nor did Slit-C accumulate on the Fas2 positive axons (arrowhead; n=2). (G) *slit*^{2} homozygous mutants had a characteristic collapse of all axons onto the midline. (H) Expression of *UAS-slit-N* in midline glia using *sim-Gal4* in a *slit*^{2} homozygous background caused a characteristic collapse of axons onto the midline, with the Slit-N fragment unable to rescue the *slit*^{2} phenotype (n=8). (I) Expressing Slit-C at the midline using a *UAS-slit-C* with *sim-Gal4* driver in a *slit*^{2} mutant background did not rescue midline repulsion (n=8).

Development • Supplementary information

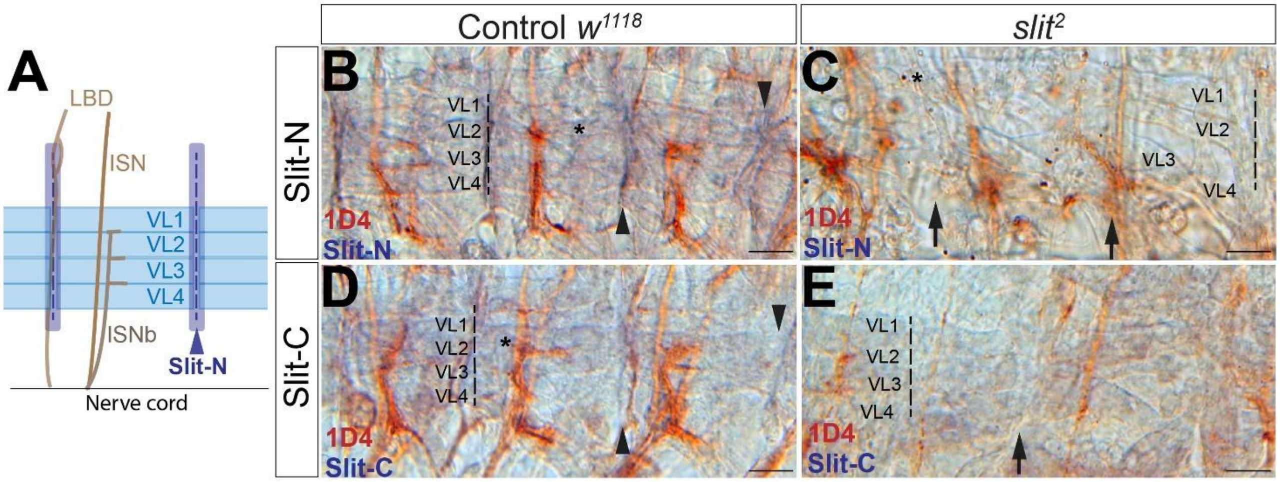

Figure S4. Differences in Slit-N and Slit-C localization in abdominal muscles. (A) Diagram of abdominal muscles adjacent to the ventral nerve cord. The ventrolateral (VL) muscles 1-4 span across an abdominal segment, denoted by dotted lines. In abdominal segments, the lateral bipolar dendritic neuron (LBD) extends along the anterior segment boundary. The intersegmental nerve branch (ISN) and intersegmental nerve branch b (ISNb) bisect the VL muscles, with the ISNb synapsing at several sites among the VL muscles. Slit-N appears localized to the intersegmental muscle attachment sites (purple, arrowhead). (B-E) Visualization of Slit localization in VL muscles with co-labels of motor neurons (LBD, ISN, ISNb, and others) with Fas2 antibody (HRP, brown) and either anti-Slit-N (B-C) or anti-Slit-C (D-E; $NiCl_2$, purple). Example segment boundaries and VL muscles are labeled in each image with dotted lines and VL1-4, respectively. All embryos are stage 17 and were treated under identical conditions. Anterior is to the left in all images, bar indicates 10μm. HRP labeling was favored over fluorescent labels as the Fas2 fluorescent signal increases signal from the muscle relative to the muscle attachment site. (B) Control *w*^{1118} embryos have a small amount of diffuse Slit-N labeling within the muscles (asterisk), and a concentrated amount of Slit-N at the muscle attachment sites (arrowheads; n=4). (C) *slit*^{2} homozygous mutants have no Slit-N labeling at the muscle attachment sites (arrows), and obvious muscle and motor neuron defects. Note that errors in muscle attachment and motor neuron guidance can make visualizing the segment boundary difficult, but the entire embryo lacks Slit-N labeling. Additionally, some DAB precipitate is visible as random dots (asterisk; n=3). (D) Control *w*^{1118} embryos labeled with Slit-C show a small amount of diffuse labeling in the VL muscles (asterisk), but no accumulation of Slit-C at the muscle attachment sites (arrowheads; n=3). (E) *slit*^{2} homozygous mutants have no Slit-C labeling in the muscles or at the muscle attachment sites (arrow; n=3).

Development • Supplementary information

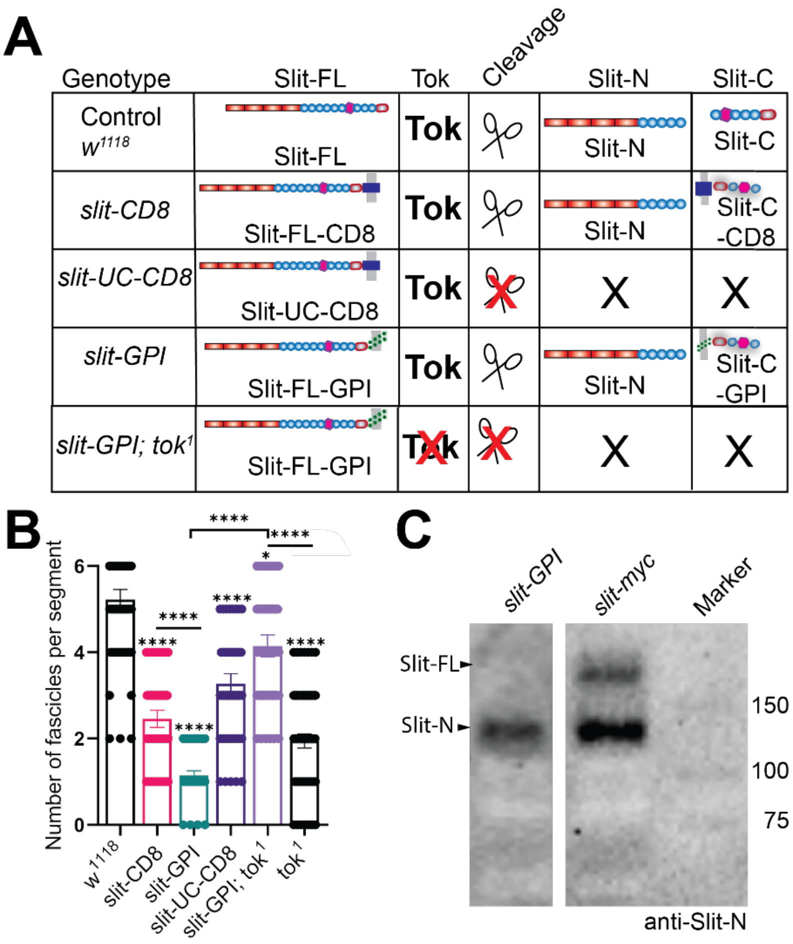

Figure S5. Additional information on epistatic interactions between *tok* and membrane-tethered *slit*. (A) Diagram depicting the state of Slit, Tok, and Slit cleavage in each genotype used in Fig. 4. Wild-type embryos have normal Slit production, resulting in circulating Slit-FL, and functioning Tok, which generates Slit-N and Slit-C fragments. Anchoring Slit via -CD8 or -GPI results in anchored Slit-FL, Slit-N, and anchored Slit-C. Removal of the Slit cleavage site (Slit-UC) or Tok both result in no Slit fragments generated, and only Slit-FL present and anchored to the cell membrane. (B) Quantification of the number of fascicles per genotype from Fig. 4 with a Kruskal-Wallis one-way ANOVA (* $p<0.05$; **** $p<0.0001$). Asterisks over bars indicate comparisons to w^{1118}, with select pairwise comparisons between *slit-GPI* and *slit-GPI*; tok^1 shown with horizontal bar. Wild-type repulsion and fasciculation are represented by ~6 fascicles per segment, while complete collapse of axons results in 1-2 fascicles per segment. Midline repulsion was significantly improved from the *slit-GPI* midline collapse in *slit-GPI;* tok^1 embryos. (C) Western blot of *slit-GPI* homozygous whole-embryo lysates showed production of Slit-N, but no Slit-FL when labeled with Slit-N antibody, indicative of hyperactive Slit cleavage (n=2). This was in contrast to wildtype embryos and *tok* mutant embryos (Fig. 1B), and *slit-myc* mutants, which have both Slit-N and Slit-FL. Note, these two samples were run on the same blot, but not in adjacent lanes.

Development • Supplementary information

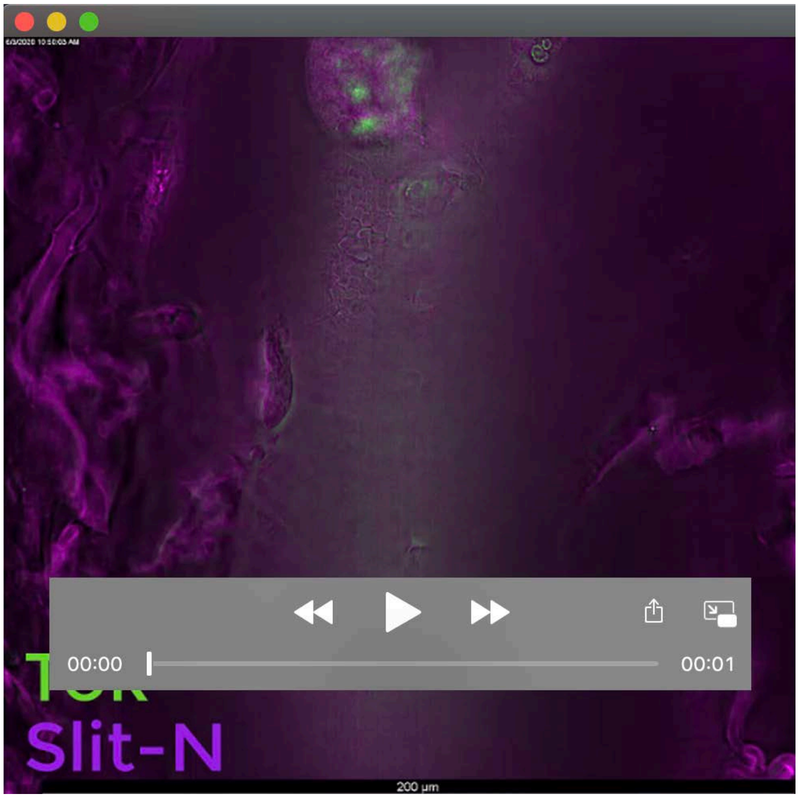

Movie 1. Tok and Slit-N expression are non-overlapping. Z-stack video of the embryo from Fig. 2A-C of Tok::GFP labeled with anti-GFP (green) and anti-Slit-N (magenta). Note that Tok::GFP appeared in specific motor neurons, in motor axons (arrows), and in underlying cell bodies, while Slit primarily labeled underlying midline glia (arrowhead). Movie comprises 134 images, with each Z slice 211.2 µm thick collected using a Leica Tissue Imager wide-field microscope with Thunder deconvolution processing. Bar indicates 200µm.
